# Metallic Castings

**Published:** 1850-01

**Authors:** 


From the N. Y. Dental Recorder.

### METALLIC CASTINGS.

In the last number of the Recorder, we published the report of the committee appointed by the Society of Dental Surgeons of the State of New York, to examine and report upon the best materials for making dentist's metallic casts. That committee consisted of five members, who, after a laborious and protracted examination of all the most common metals and alloys used by dentists, finally agreed in recommending an alloy of zinc and tin as combining more useful properties for this purpose than any other. Since that time we have been politely furnished by one of the committee, (Dr. Geo. E. Hawes,) with the result of his experiments, which will be found both interesting and instructive to all practical dentists laboring in this department of the art.

One of the principal objects of this committee was to find a metal or alloy from which suitable castings could be made with the least possible shrinkage. To ascertain the different degrees which the various metals and alloys contracted while crystalizing and cooling, Dr. Hawes constructed a very simple and ingenious instrument, similar to Dr. Hare's pyrometer. From a pattern, one foot in length, bars of all the different metals which he wished to test were cast, being moulded with care by an experienced workman, in sand, and cast at the lowest degree of temperature at which the metal could be poured.

To test the hardness and tenacity of the different metals, Dr. Hawes made use of the jeweller's drop. After casting a bullet from each, it was placed in the drop, the weight raised to a certain height, and then suffered to fall on the bullet. The hardness of the metal would evidently be shown by the distance to which the bullet was flattened, while the fracture or cracks around the edge would indicate different degrees of tenacity.

TABLE OF THE CONTRACTION OF METALS.

| | | | | |
|---|---|---|---|---|
| 1. Covill's Metal | expands slightly, | 1.64 | inch | per foot. |
| 2- Jones' " | contracts | 1.32 | " | " |
| 3. Britannia " | " | 1.28 | " | " |

| | | | |
|---|---|---|---|
| 4. | Tin metal contracts | | 1.24 inch per foot. |
| 5. | Type " " | | 1.20 " " |
| 6. | Zinc 1 lb. to 6 oz. tin, contracts | | 1.16 " " |
| 7. | " 1 " to 1 " " " | about | 1.12 " " |
| 8. | Lead contracts | " | 1.10 or 7.64 " |
| 9. | Iron " | " | 1.8 or 9.128 " |
| 10. | Zinc " | " | 1.8 or 11.128 " |
| 11. | Brass " | " | 3.16— " |

The following recipes were given to the committee during their investigation, and all of them contain some good properties. We give them with the results of Dr. Hawes' observations and experiments upon them:

MR. S. COVILL'S RECIPE.

| | |
|---|---|
| Tin, | 5 parts. |
| Bismuth, | 3 " |
| Lead, | 2 " |
| Mercury, | 1 " |

This composition expands slightly, about 1.64 of an inch to a foot—is brittle and rather soft. It will probably require two dies, one to form the plate, and the other to perfect it. A bullet placed under the weight in a jeweller's drop was broken to pieces.

MR. GEO. CLAY'S RECIPE.

| | |
|---|---|
| Tin, | 3 ounces. |
| Antimony, | 3 " |
| Zinc, | 3 1-4 " |
| Bismuth, | 1 1-2 " |
| Copper, | 1 1-2 " |

This composition makes a good die, which may be dipped into lead to form the matrix. It must be cast as soon as melted, or its proportions would be changed by the oxydation of some of the more fusible metals.

FUSIBLE METALS.

| | |
|---|---|
| Tin, | 5 parts. |
| Bismuth, | 3 " |
| Mercury, | 1 " |

From this metal the die is cast; and to form the matrix add 1 oz. of mercury to 4 lbs. of the above. The principal recommendation of this metal is the facility with which it may be used, after a little practice. To the wax impression, add a border of the same material an inch and a half high, place it in the bottom of a basin and fill with cold water very near to the top of the wax border. The melted metal may now be poured directly into the wax, and should be cooled as soon as possible, by throwing the cold water immediately over it. The metal for the matrix should be melted in a strong wrought-iron dish, and kept in it while striking up the plate to prevent it from cracking. A block of hard wood should also be placed over the die to strike upon, for the same reason. This metal would not resist the blow when placed in the drop, but flew into a thousand pieces. It fuses at about 140 deg. Fahrenheit.*

ZINC AND TIN.

Many experiments were tried by Dr. Hawes with these two metals in different proportions, from 1 to 7 oz. of tin to a pound of zinc. With the increase of the tin, the shrinkage becomes less, gradually approximating to that of pure tin, which contracts about one-third as much as zinc, and fusing at a lower degree of temperature with every addition of tin. At the point of six or seven ounces of tin to a pound of zinc, the alloy became too soft for use. The committee finally concurred in re-

* The following recipes for fusible metal are taken from the last number of the Dental News Letter.

*Fusible Metal.*—The following recipe has been sold to some of the dentists, and is said to be a valuable one. We paid five dollars for it, and thus give it to the whole profession.

| NO. 1, OR HARD. | | NO. 2, OR SOFT. | |
|---|---|---|---|
| Bismuth, | 8 parts. | Bismuth, | 8 1-2 parts. |
| Lead, | 5 " | Lead, | 5 1-4 " |
| Block Tin, | 3 " | Block Tin, | 3 1-4 " |
| Mercury, | 2 " | Mercury, | 2 3-8 " |

No. 1 is for the male. No. 2 for the female cast. The lead should be melted first, then add the tin, and when they are well melted, pour the bismuth into the lead and tin. (The bismuth should be ready melted in another vessel.) The mercury should be added slowly. None of the metals should be hotter than just sufficient to amalgamate.

This preparation is poured into the wax impression, which was previously hardened, thus saving the trouble of taking plaster models, and of moulding in sand.

commending to dentists an alloy of three parts of zinc to one of tin, as, all things considered, the best adapted for metalic castings of any metal or composition with which they were acquainted.

